# RETRACTION: Challenges and Opportunities of Big Data Analytics in Healthcare

**DOI:** 10.1002/hcs2.70006

**Published:** 2025-03-25

## Abstract

**RETRACTION:** P. Goyal and R. Malviya, “Challenges and Opportunities of Big Data Analytics in Healthcare,” *Health Care Science* 2, no. 5 (2023): 328–338, https://doi.org/10.1002/hcs2.66.

The above article, published online on 4 October 2023 in Wiley Online Library (wileyonlinelibrary.com), has been retracted by agreement between the journal Editor‐in‐Chief, Zongjiu Zhang; Tsinghua University Press; and John Wiley & Sons Ltd. The retraction has been agreed due to unattributed overlap between this article and a previously published article [1]. The corresponding author R. Malviya disagrees with the retraction. P. Goyal was informed of the decision to retract but remained unresponsive.
